# Comment on “Acute Sarcopenia: Systematic Review and Meta‐Analysis on Its Incidence and Muscle Parameter Shifts During Hospitalisation” by Aldrich et al.

**DOI:** 10.1002/jcsm.13767

**Published:** 2025-03-05

**Authors:** Paulo Eugênio Silva, Gerson Cipriano

**Affiliations:** ^1^ Department of Physical Medicine and Rehabilitation University of São Paulo São Paulo Brazil; ^2^ Intensive Care Unit of Hospital de Base do Distrito Federal (IGESDF) Brasília Brazil; ^3^ Rehabilitation Sciences Program (PPGCR), Faculty of Ceilândia University of Brasília Brasília Brazil

Dear Editor,

We are writing to address a misrepresentation of our study, Silva et al. [1], in the recently published article titled ‘Acute Sarcopenia: Systematic Review and Meta‐Analysis on Its Incidence and Muscle Parameter Shifts During Hospitalisation’ [2]. Specifically, the authors state on page 11 that Silva et al. [1] did not measure knee extension strength. This statement is incorrect.

In our study, knee extension strength was assessed using a dynamometer to measure peak force evoked by neuromuscular electrical stimulation. This methodology was a fundamental part of our research design and is thoroughly detailed in the published manuscript [1] as well as in other related manuscripts [3, 4]. Please refer to Figure 1 and method section below described.

**FIGURE 1:**
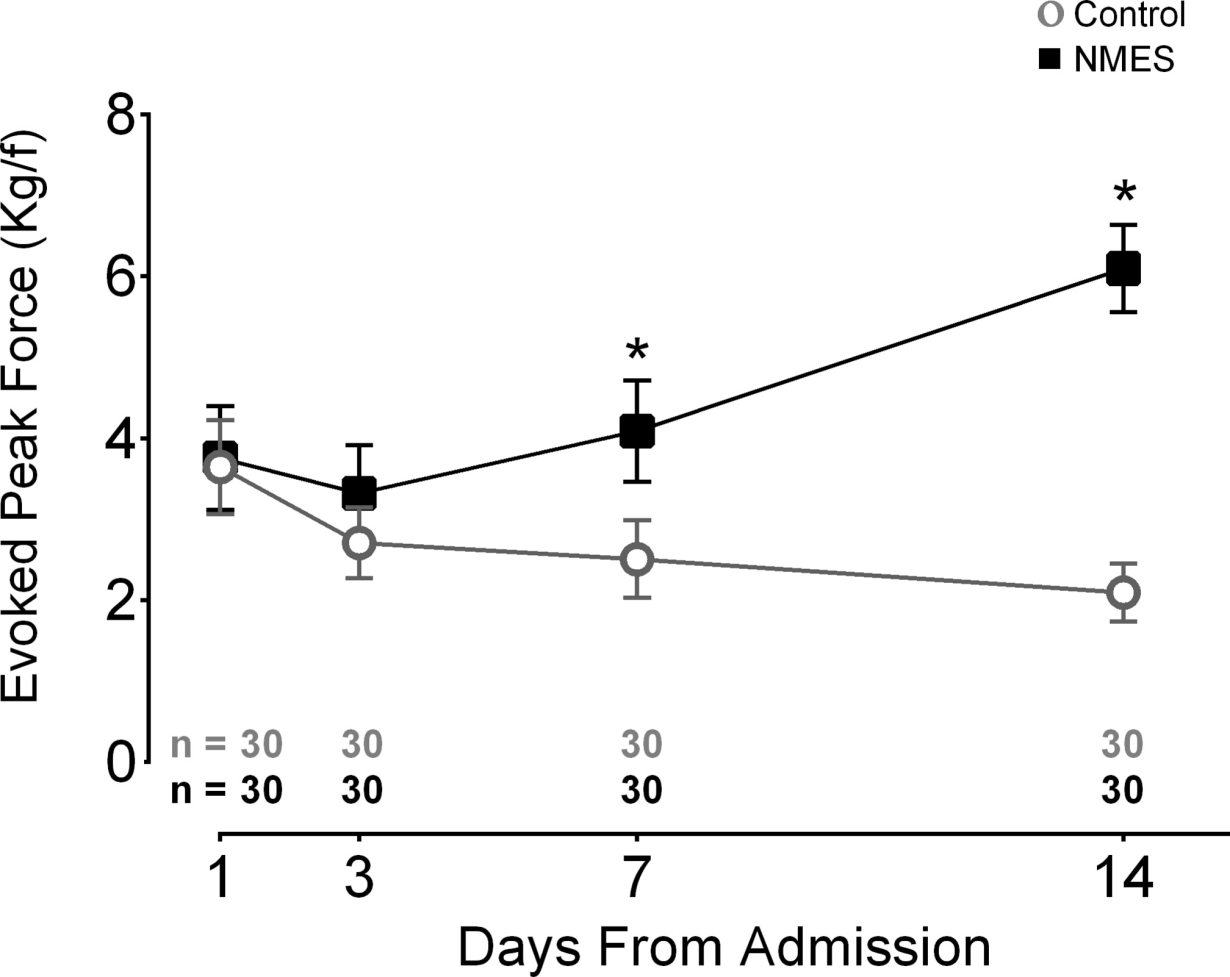
Effect of bed rest time and NMES on electrically evoked peak force. This graph presents the electrically evoked peak force of the rectus femoris muscle. The highest value after three bouts of electrical stimuli is reported. The contraction was elicited with a pulse duration and frequency of 400 μs and 100 Hz, respectively with 69‐mA amplitude and 3 s of time on. Two electrocardiogram electrodes were placed over the rectus femoris motor points. Kgf: kilogram force; *: statistically significant time × group effect on highlighted day. This effect was analysed by repeated measures two‐way ANOVA. An intention‐to‐treat analysis was performed for all randomized participants. This figure was originally Figure 4 that was extracted from Silva et al. [1, p. 9].

Methods section from Silva et al. 2019 [1].

To evaluate the evoked peak force, we used a calibrated load cell (CKS model, Kratos Equipamentos, São Paulo, Brazil) attached to a platform and an electrical stimulator (Dualpex 071, Quark Medical, Brazil). Patients were laid down in a supine position with a 30° bed elevation. The platform was adjusted to the hip position at 90° of flexion and knee at 60° of the extension where the highest torque occurs. The electrodes used to evoke muscle contraction were positioned on the rectus femoris muscle. The stimuli were performed on twitch contraction with 69 mA, Time on of 3 seconds, pulse width, and frequency of 400 μs and 100 Hz respectively. Three stimuli were performed, and the interval between each measurement was 2 min. We used the highest detected value among the measures. [1]

Additionally, we would like to clarify a discrepancy regarding the sample size reported for our study. The correct sample size was 60 participants (Figure 2), not 30 as might be inferred from the tables and figures in our manuscript, specifically Figures 1 and 2.

**FIGURE 2:**
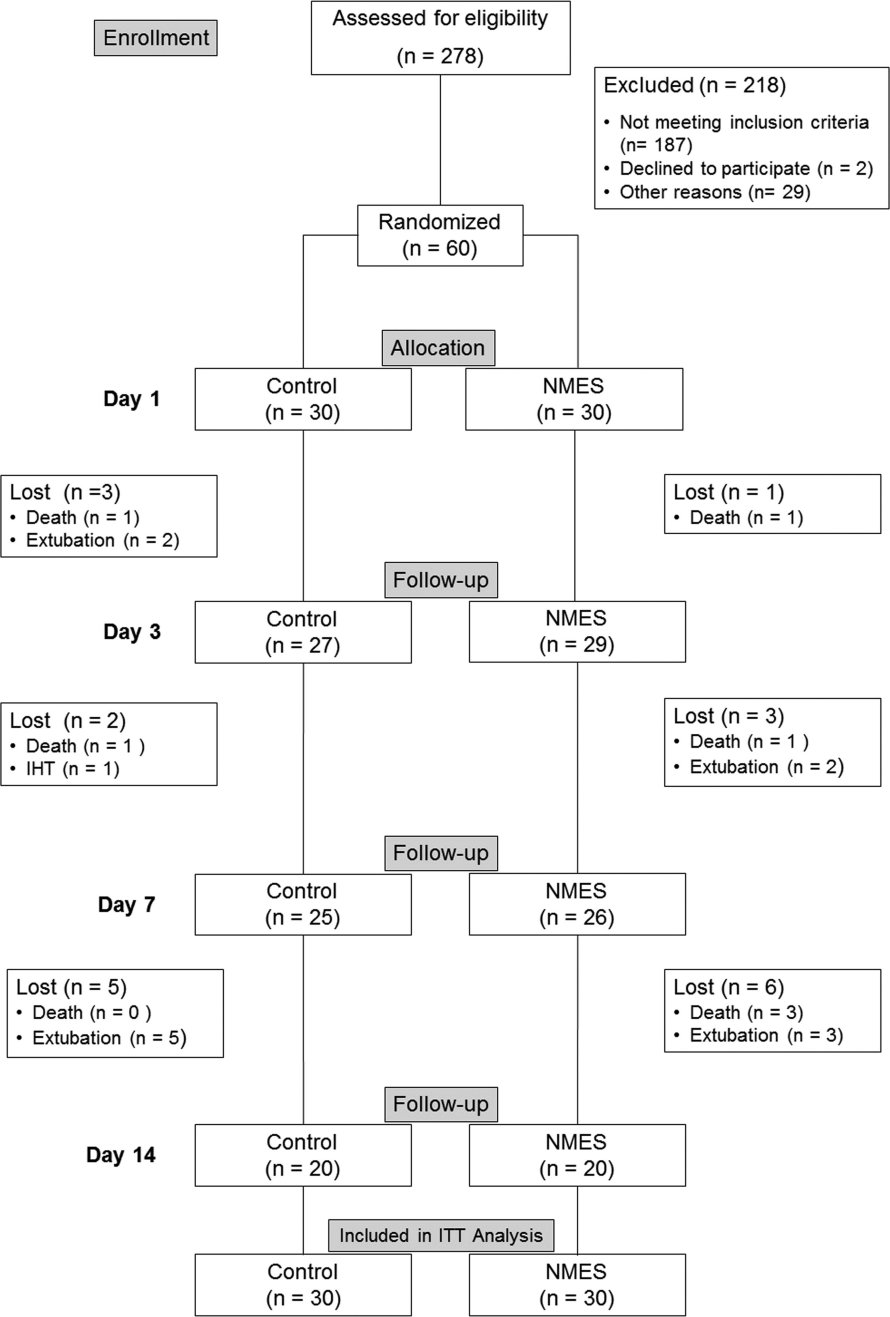
Consort diagram. IHT: inter‐hospital transfers; ITT: intention‐to‐treat; other reasons: technical problems, death before randomization and inter‐hospital transfers. This figure was originally Figure 1 that was extracted from Silva et al. [1, p. 5].

It appears that the authors may have considered only the control group, but this is not explicitly stated. It is important for readers to have accurate information about the study design and sample size to properly interpret the findings.

We kindly request that these inaccuracies be addressed to ensure the integrity of the scientific record and to prevent potential misinterpretations by readers and researchers relying on this review.

## Ethics Statement

The authors have nothing to report.

## Conflicts of Interest

The authors declare no conflicts of interest.

## Data Availability

The authors have nothing to report.
